# Benthic macroinvertebrates assemblages of glacial-fed (Bheri) and rain-fed (Babai) rivers in western Nepal in the wake of proposedinter-basin water transfer

**DOI:** 10.3897/BDJ.10.e79275

**Published:** 2022-02-07

**Authors:** Kumar Khatri, Smriti Gurung, Bibhuti Ranjan Jha, Udhab Raj Khadka

**Affiliations:** 1 Mahendra Ratna Campus, Tribhuvan University, Kathmandu, Nepal Mahendra Ratna Campus, Tribhuvan University Kathmandu Nepal; 2 Department of Environmental Science and Engineering, Kathmandu University, Dhulikhel, Nepal Department of Environmental Science and Engineering, Kathmandu University Dhulikhel Nepal; 3 Central Department of Environmental Science, Tribhuvan University, Kirtipur, Nepal Central Department of Environmental Science, Tribhuvan University Kirtipur Nepal

**Keywords:** macroinvertebrates, glacial-fed river, rain-fed river, inter-basin water transfer, inventory, Nepal

## Abstract

**Background:**

Benthic macroinvertebrates, encompassing large taxonomic groups of invertebrate organisms, are important components of aquatic ecosystems and play crucial roles in aquatic food webs. These organisms are also extensively used in water quality assessments as bioindicators for a range of stressors. Inter-basin water transfer (IBWT) is the transfer of water from a donor basin to a recipient basin and such transfers have both beneficial as well as adverse environmental and socio-economic impacts. This study attempts to generate baseline information on macroinvertebrates assemblages in glacial-fed (Bheri) and rain-fed (Babai) rivers of west Nepal, where Nepal’s first ever inter-basin transfer is in progress and the data can be used to assess the impact of inter-basin water transfer on water quality and aquatic biodiversity after completion.

**New information:**

The dataset includes the records on the taxonomic diversity of macroinvertebrate in the Bheri and the Babai River systems. A total of 56 families of macroinvertebrates belonging to eight classes and four phyla were observed. A significant variation between the glacial-fed and rain-fed and seasons were observed reflecting different ecological zones and abiotic variables in the rivers and their catchments. Hydropsychidae and Baetidae were reported to be the most abundant taxa in the Bheri River system, whereas Gyrinidae, Physidae, Chironomidae and Hydropsychidae were most abundant taxa in the Babai River system.

## Introduction

Benthic macroinvertebrates encompass a rich taxonomy and are widely distributed and found abundantly in water bodies (Benetti et al. 2012). They play crucial roles in aquatic food webs and maintain ecological integrity of aquatic ecosystems. Macroinvertebrates are primary consumers as they are the primary processors of organic materials and thus, play a key role in nutrient cycling in aquatic ecosystems (Covich et al. 1999). In addition to their role as primary consumers, they also serve as detritivores consuming decomposing organic matter and also act as predators (Carter et al. 2017). These groups of organisms are sensitive to even small changes in physico-chemical parameters in water bodies and their catchments (Ganguly et al. 2018). They respond to different stream conditions and levels of stressors and their presence or absence can be used to indicate the impact of such stressors (Labajo-Villantes and Nuñeza 2015). Accordingly, they are one of the most commonly-used bioindicators of water bodies (Rosenberg 1993).

Freshwater resources are one of the most impacted ecosystems (Pereira et al. 2009) as humans have explored and developed several ways to minimise the issue of water scarcity, such as recycling wastewater, seawater desalination, virtual water trade, inter-basin water transfer, rain water harvesting technology and restoration of wetlands (Zhuang 2016). One of the effective ways to minimise water scarcity problems and to balance the water in deficit and surplus areas is the concept of inter-basin water transfer (IBWT) which involves transfer of water from water-surplus basins to water-deficient basins (Tien Bui et al. 2020). Although such projects have significant benefits, they also tend to have adverse upstream and downstream impacts and may affect river morphology, river flow, hydrologic systems, water quality, vegetation and biota in both donor and the recipient basins (Tien Bui et al. 2020). Such alterations and modifications have potential implications on biotic communities, such as changes in alteration of aquatic habitats, introduction of non-indigenous organisms and impacts on migratory species (Cole Sr and Carver 2011). Apart from this, such transfers are also known to have socio-economic implications (Rangachari 2005, White 2014). The Eastern National Water Carrier, the Tugela-Vaal Water Transfer Scheme in southern Africa, Snowy Mountains Scheme in south-east Australia etc. provide examples of the adverse impacts of inter-basin transfers on aquatic biota, such as the macroinvertebrates, algae and fishes (Davies et al. 1992, Snaddon et al. 1999, Bunn and Arthington 2002). Therefore, impact assessment of IBWT projects becomes crucial. In order to assess the impacts of IBWT, baseline information on physical, chemical and biological parameters are required which can act as reference for future impact assessment studies.

## Sampling methods

### Sampling description

Macroinvertebrate sampling followed the microhabitat approach (Barbour et al. 1999). Different microhabitats like riffles, pools and runs were considered during sampling. A standard 250 micrometer (μm) mesh net was used to sample the macroinvertebrates. The net was placed against the flow of the river and the substrates were disturbed with the heels of the feet. The dislodged macroinvertebrates, carried downstream by the water current, were trapped in the net. At each site, 50–100 m stretch was considered and 10 subsamples were collected to form a composite sample. Larger substrates were picked up and rubbed by hand to remove attached organisms. Each composite sample was transferred into a collecting jar, preserved in 70% ethanol and all samples were brought to the laboratory of the Department of Environmental Science and Engineering (DESE), Kathmandu University for identification.

### Step description

Macroinvertebrates were sorted and identified to family level by following standard literature (Merritt and Cummins 1996, Dudgeon 1999) and regional keys.

## Geographic coverage

### Description

**Figure 3.**
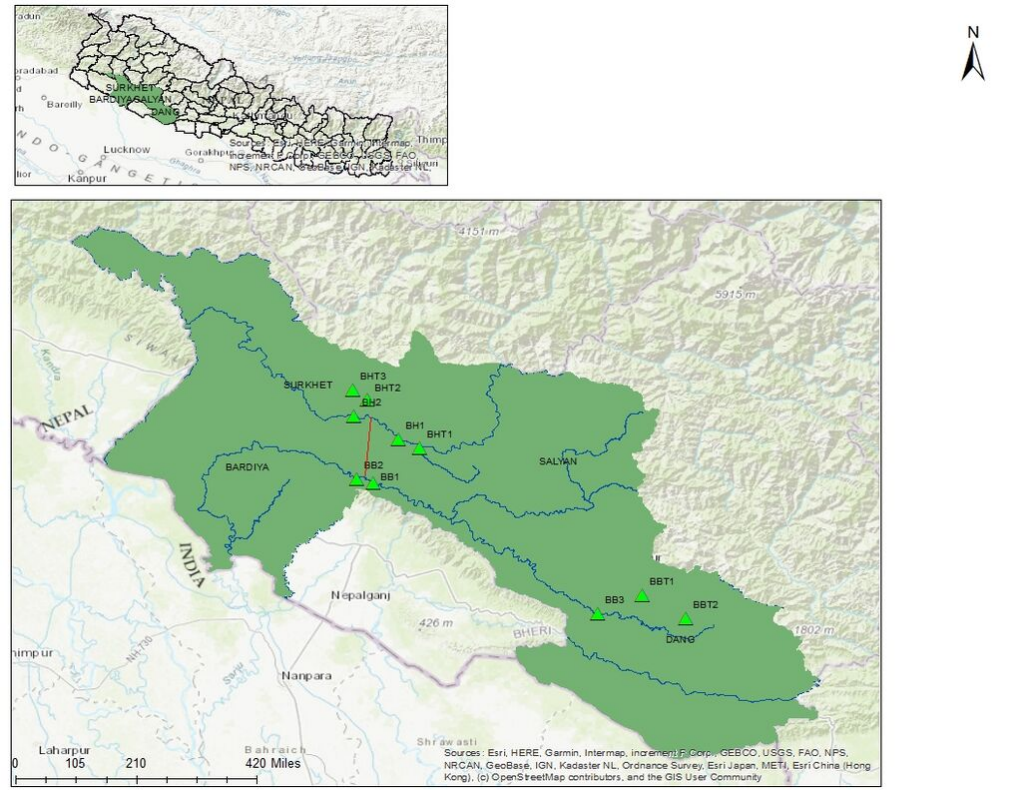
The map showing the sampling locations.

The study was conducted in the Bheri and the Babai Rivers and some of their tributaries in the wake of proposedfirst inter-basin water transfer in western Nepal. The project aims to divert 40 m^3^/s of water from Bheri River to Babai River through a 12.2 km long tunnel to achieve yearround irrigation for 51000 ha of agricultural land in Banke and Bardiya districts and to generate 46 MW electricity (GoN/DoWRI 2018). The Bheri River is a major tributary of the Karnali River, located in western Nepal and originates in the glacier of the Himalayas (Negi 1991). The average discharge of the Bheri River at Samaijighat (Station No. 269.5 located at 500 m a.s.l.), upstream of the proposed water diversion, during summer is 805 ± 635.20 m^3^/s; 346.70 ± 369.40 m^3^/s in autumn; 86.82 ± 6.22 m^3^/s in winter and 82.67 ± 7.59 m^3^/s in spring (GoN/DoHD 2019). The Babai is a perennial river, which originates in the Mahabharat Range, is fed by springs, precipitation and groundwater, but the volume of water is low during the dry season (Sharma 1977). The average discharge of the Babai River at Chepang (Station No. 289.95 located at 325 m a.s.l.), near the proposed release, during summer is 75.84 ± 94.17 m^3^/s; 42.06 ± 38.68 m^3^/s in autumn; 1.45 ± 1.01 m^3^/s in winter and 8.91 ± 6.27 m^3^/s in spring (GoN/DoHD 2019). Upstream and downstream of the water diversion at Bheri and upstream and downstream of the water release at Babai, three upstream tributaries of Bheri and three upstream sites (two tributaries and one mainstream) of Babai were sampled. Therefore, a total of 10 sites were sampled for this study (Figs 1, 2, 3, Table 1). Since one of the sampling sites - Mulghat - is located in Bardiya National Park, a mandatory permit for sampling was taken from the Department of National Park and Wildlife Conservation (DNPWC).

## Taxonomic coverage

### Description

The dataset includes the records on the taxonomic diversity of macroinvertebrates in the Bheri and the Babai River systems. A total of 56 families of macroinvertebrates belonging to eight classes and four phyla were observed. Hydropsychidae (during winter and summer) and Baetidae (during spring and autumn) were reported to be the most abundant taxa in the Bheri River system, whereas Gyrinidae (during winter), Physidae (during summer), Chironomidae (during autumn) and Hydropsychidae (during spring) were the most abundant taxa in the Babai River system (Fig. 4).

### Taxa included

**Table:** 

Rank	Scientific Name	
phylum	Arthropoda	
phylum	Platyhelminthes	
phylum	Mollusca	
phylum	Annelida	
class	Insecta	
class	Bivalvia	
class	Gastropoda	
class	Malacostraca	
class	Turbellaria	
class	Arachnida	
class	Clitellata	
class	Polychaeta	
order	Ephemeroptera	
order	Odonata	
order	Plecoptera	
order	Hemiptera	
order	Megaloptera	
order	Coleoptera	
order	Trichoptera	
order	Lepidoptera	
order	Diptera	
order	Sphaeriida	
order	Basommatophora	
order	Neotaenioglossa	
order	Decapoda	
order	Tricladida	
order	Acarina	
order	Oligochaeta	
order	Haplotaxida	
order	Opisthopora	
order	Phyllodocida	
family	Ameletidae	
family	Baetidae	
family	Heptageniidae	
family	Arthropleidae	
family	Ephemerellidae	
family	Caenidae	
family	Leptophlebiidae	
family	Potamanthidae	
family	Ephemeridae	
family	Gomphidae	
family	Macromiidae	
family	Libellulidae	
family	Calopterygidae	
family	Euphaeidae	
family	Synlestidae	
family	Perlidae	
family	Veliidae	
family	Gerridae	
family	Nepidae	
family	Aphelocheiridae	
family	Micronectidae	
family	Corydalidae	
family	Gyrinidae	
family	Dytiscidae	
family	Hydrophilidae	
family	Psephenidae	
family	Elmidae	
family	Hydropsychidae	
family	Philopotamidae	
family	Polycentropodidae	
family	Psychomyiidae	
family	Glossosomatidae	
family	Hydroptilidae	
family	Rhyacophilidae	
family	Brachycentridae	
family	Stenopsychidae	
family	Pyralidae	
family	Ceratopogonidae	
family	Athericidae	
family	Empididae	
family	Tabanidae	
family	Limoniidae	
family	Culicidae	
family	Simuliidae	
family	Chironomidae	
family	Sphaeriidae	
family	Physidae	
family	Planorbidae	
family	Thiaridae	
family	Palaemonidae	
family	Planariidae	
family	Acaridae	
family	Oligochaeta indet.	
family	Naididae	
family	Megascolecidae	
family	Nereididae	

## Temporal coverage

### Notes

In order to generate baseline information on macroinvertebrates in the wake of proposedinter-basin transfer from glacial-fed Bheri to rain-fed Babai River systems, a macro-invertebrate inventory was generated. In this paper, we provide the family level dataset of macroinvertebrates covering a period of one year (2018) encompassing four seasons, namely winter (January), spring (March-April), summer (June) and autumn (October).

## Collection data

### Collection name

Benthic macroinvertebrates of the Bheri and the Babai River systems.

### Specimen preservation method

70% Ethanol

## Usage licence

### Usage licence

Creative Commons Public Domain Waiver (CC-Zero)

## Data resources

### Data package title

Benthic macroinvertebrates data of the Bheri and the Babai River systems.

### Number of data sets

1

### Data set 1.

#### Data set name

Benthic macroinvertebrates data of the Bheri and the Babai River systems

#### Number of columns

14

#### Data format version

CSV (comma delimited)

#### Description

The presented data (Suppl. material 1) contains information on the distribution and species composition of benthic macroinvertebrates in the Bheri and the Babai River systems in western Nepal.

**Data set 1.** 

Column label	Column description
River	Name of River
Site Code	Code assigned to sampling sites
Place	Place of sampling
Elevation	Metres above sea level (m a.s.l.)
Longitude	Longitude (ºN)
Latitude	Latitude (ºE)
District	Name of District
Date	Date of sampling
Seasons	Name of seasons
Phylum	Macroinvertebrate Phylum
Class	Macroinvertebrate Class
Order	Macroinvertebrate Order
Family	Macroinvertebrate Family
Total Catch	Total number of Macroinvertebrates trapped

## Additional information

Macroinvertebrate assemblages:

A total of 21,866 macroinvertebrates belonging to 56 families, 8 classes and 19 orders were captured during the sampling periods. A total of 11,473 macroinvertebrates contributing 52.47% belonging to 42 families and 13 orders were observed in the Bheri River system, whereas a total of 10,393 macroinvertebrates contributing 47.53% belonging to 49 families and 18 orders were observed in the Babai River system. Insect fauna represented the bulk of macroinvertebrate communities in both river systems.

In the Bheri River system, 38 families of insects were observed contributing 99.41%, whereas in the Babai River system, 39 families of insects were observed contributing 79.77% of total macroinvertebrate fauna. The non-insect fauna in the Babai system were represented by 10 families with four Mollusca taxa; one each of Malacostraca and Arachnida taxa and; four Annelida taxa. A number of studies in Nepal have reported insects as dominant macroinvertebrates in streams and rivers (Matangulu et al. 2017, Rai et al. 2019, Shah et al. 2020, Gurung et al. 2021). Non-insect fauna, particularly Mollusca, have been reported in lowland water bodies (Nesemann and Sharma 2005) as opposed to mid-hill rivers and streams. Insects are the most speciose species (Samways 1993) and most of the insects spend their larval or nymphal stages in aquatic ecosystems (Merritt et al. 2019). The %EPT (Ephemeroptera, Plecoptera and Trichoptera) was also higher in the Bheri River system (74.15%) than in the Babai River system (34.11%) and this finding is in accordance with previous studies in eastern (Gurung et al. 2021), mid-western (Sharma et al. 2015) and far-west (Suren 1994) Nepal and elsewhere (Mishra and Nautiyal 2013). Ephemeroptera, Plecoptera, Trichoptera and Diptera are considered as major benthic communities of freshwaters (Malicky et al. 2008, Nautiyal et al. 2013).

In the Bheri River system the highest number of macroinvertebrates were found in site BH2 (1642 individuals during the spring season), while the lowest number of macroinvertebrates was also observed in BH2 (only 123 individuals during the autumn season). In the Babai River system, the highest number of macroinvertebrates were found in BB3 (1266 individuals during the autumn season), while the lowest number of macroinvertebrates were observed in BB1 (186 individuals during the winter season). The most dominant taxa and the taxa with the lowest number of individuals (only 2) in different seasons in both the river systems are listed in Table 2. The Mann Whitney U test revealed significant variation (p < 0.05) in the abundance of Baetidae, Heptageniidae, Ephemerellidae, Ephemeridae, Psephenidae, Stenopsychidae, Chironomidae and Physidae between the Bheri and the Babai River systems.

Baetidae is a common taxon and occurs in almost all freshwater habitats including fast flowing riffles, runs, pools and wetlands, but they are most diverse in cool flowing water (Dean and Suter 1996). Baetidae, Simuliidae and Chironomidae are quite common taxa in freshwater and are known to be present even in disturbance zones (Mackay 1992). Baetidae and Hydropsychidae have also been reported to be common taxa from many Nepalese rivers (Ormerod et al. 1994, Suren 1994, Gurung et al. 2013, Bhandari et al. 2018). Hydropsychidae are filter feeders (Cushing Jr 1963) and their abundance in most of the lotic systems in Nepal indicates presence of sediment in rivers. Babai with its low flow compared to Bheri, warmer water temperature and its catchment more prone to soil erosion and landslides after intense rainfall (Bhandari and Dhakal 2018) might have favoured food availability and habitat for Hydropsychidae. Physidae is an air breather (Koopman et al. 2016), so they can even survive in low dissolved oxygen (DO). Physidae prefer slow moving shallow water and they feed on algae, diatoms, other organic wastes and macrophytes and their abundance is higher in warm waters. At the time of sampling, the tributaries of Babai were characterised by low flow compared to others and were filled with algae. The warm water, algal growth and relatively lower DO (5.53 mg/l) might explain the abundance of Physidae in the tributaries of the Babai River system.

The Jaccard distance value was 0.37 indicating that 37% of the macroinvertebrate taxa were dissimilar between these two river systems. Taxa such as Baetidae, Ephemerellidae, Caenidae, Leptophlebiidae, Gomphidae, Perlidae, Philopotamidae, Tabanidae, Limoniidae and Chironomidae were observed in both the river systems and during all four seasons. Families like Arthropleidae, Potamanthidae, Polycentropodidae, Glossosomatidae, Rhyacophilidae, Empididae and Planariidae were found exclusively in the Bheri River system, whereas Families like Libellulidae, Calopterygidae, Synlestidae, Nepidae, Dytiscidae, Hydroptilidae, Ceratopogonidae, Culicidae, Sphaeriidae, Planorbidae, Thiaridae, Naididae, Megascolecidae and Nereididae were exclusively found in the Babai River system. However, it should be noted that the taxa reported in this study only reflects those from selected sites. Oligochaetes, such as Naididae, are known to be associated with macrophytes and abundant in fine sediments (Cortelezzi et al. 2011) and the presence of macrophytes and fine sediment beds in the tributaries of the Babai supports these taxa in those streams. Similarly, Physidae and Planorbidae, which show preference to slow moving shallow water (Voigts 1976), were abundant in Babai’s tributaries. Leptophlebiidae are known to prefer warmer waters (Savage 1987) and were present in the Babai River system. In contrast, Rhyacophilidae are typical cold-water taxa requiring high dissolved oxygen concentrations (Corkum 1989) and this taxon was observed only in glacial-fed Bheri, characterised by higher flow and dissolved oxygen (11.6 mg/l).

Despite rich water resources (GoN/WECS 2011), aquatic biodiversity assessments, including those of macroinvertebrates, are still scant in the Nepalese context. A recent study in Karnali River Basin in western Nepal has reported 84 families of freshwater macroinvertebrates (Shah et al. 2020). Other studies have reported 51 families from the Bagmati River (Rai et al. 2019); 53 families from western Nepal (Suren 1994) and 49 families from eastern Nepal (Gurung et al. 2021).

### Diversity Indices

Different diversity indices, such as Shannon-Wiener diversity (H’), Pielou’s evenness (J), Simpson’s Diversity index (1-D) and species richness of sampling sites in different seasons, were calculated (Table 3). The mean Shannon-Weiner diversity was higher in the Babai River system (1.76 ± 0.49) than that of the Bheri River system (1.68 ± 0.48). The Kruskal Wallis test revealed significant variation in Shannon-Weiner diversity in seasons only in the Babai River system (H = 4.35, df = 3, p = 0.23). Taxa richness in the Bheri River system ranged from 5-23, while in the Babai River system, it ranged from 4 to 24. Taxa diversity and richness are affected by a range of environmental variables, such as hydrology, dissolved oxygen, nutrient concentrations, temperature, pH etc. (Zhuang 2016, Custodio et al. 2018). In general, diversity tends to be higher in stable environments than disturbed conditions (Odum and Barrett 1971). Family-based Shannon-Weiner diversity (H’) values of < 1 indicate poor water quality, values of 1 < H’ < 3 indicate moderate water quality and > 3 indicates good water quality (Welch and Naczk 1992). Most of the sampled sites showed H’ values of 1 < H’ < 3 indicating moderate water quality. The results of the GRSBIOS (The Ganga River System Biotic Score) also supported this finding. The majority of streams and rivers, particularly in the mid-hills and lowlands in Nepal, are impacted by agricultural runoffs (Dahal 2010). The presence of agricultural runoff and low flow, particularly during spring seasons, explains the low taxa richness in BBT2 and BHT1.

### Water Quality Class, based on GRSBIOS/ASPT

The macroinvertebrate-based ecological assessment tool, GRSBIOS (Nesemann et al. 2007) was used to assess water quality of the sampling sites. In this method, around 110 insect and non-insect taxa are given scores ranging from 1 to 10. Lower scores have been assigned to tolerant taxa, whereas higher scores have been assigned to sensitive taxa. The GRSBIOS/ASPT (GRSBIOS/ Average Score Per Taxon) was calculated by dividing the total scores assigned to macroinvertebrate taxa by the total number of the taxa present at the particular site. From the obtained GRSBIOS/ASPT value, the Water Quality Class (WQC) was calculated using the transformation table (Table 4) adapted from NEPBIOS/ASPT (Nepalese Biotic Score/Average Score Per Taxon).

Table 5 shows the Water Quality Class (WQC) at different sampling sites in different seasons. The result indicates that the water quality of most of the sites was moderate to heavily polluted. Sites BBT1 during winter and spring and BBT2 during spring and autumn were found to be more polluted than other sites. These sites were characterised by low flow, algal growth and lower DO values with large numbers of Naididae and Chironomidae. These taxa are typical of organic pollution (Küçük 2008).

### Conclusion

A total of 56 macroinvertebrate families were observed indicating a rich macroinvertebrate biodiversity in these river systems. A significant variation in macroinvertebrate assemblages between the glacial-fed and rain-fed river systems and seasons were observed reflecting different ecological zones and abiotic variables in the rivers and their catchments. The ongoing inter-basin water transfer is likely to affect different environmental variables and biota including macroinvertebrate assemblages. Therefore, baseline data of macroinvertebrates, generated from this study, would be useful as future references for impact assessment of inter-basin transfer of water from glacial-fed (Bheri) to rain-fed (Babai) rivers in western Nepal.

## Supplementary Material

C2D4E4CF-B8CB-5BE5-A08E-98F38C607A04 10.3897/BDJ.10.e79275.suppl1 Supplementary material 1 Benthic macroinvertebrates in the Bheri and the Babai River systems in western Nepal. Data type CSV (comma delimited) Brief description The presented data contain information on the distribution and species composition of benthic macroinvertebrates in the Bheri and the Babai River systems in western Nepal. File: oo_633749.csv https://binary.pensoft.net/file/633749 Kumar Khatri, Smriti Gurung, Bibhuti Ranjan Jha, Udhab Raj Khadka

## Figures and Tables

**Figure 1a.**
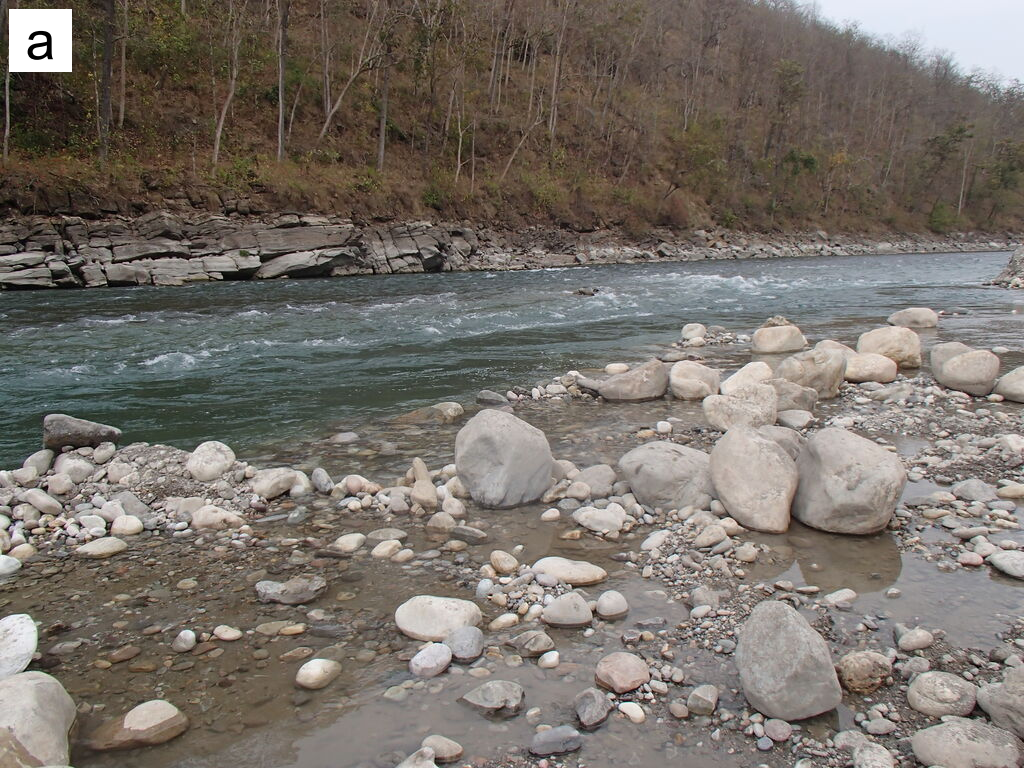
Bheri River (BH1)

**Figure 1b.**
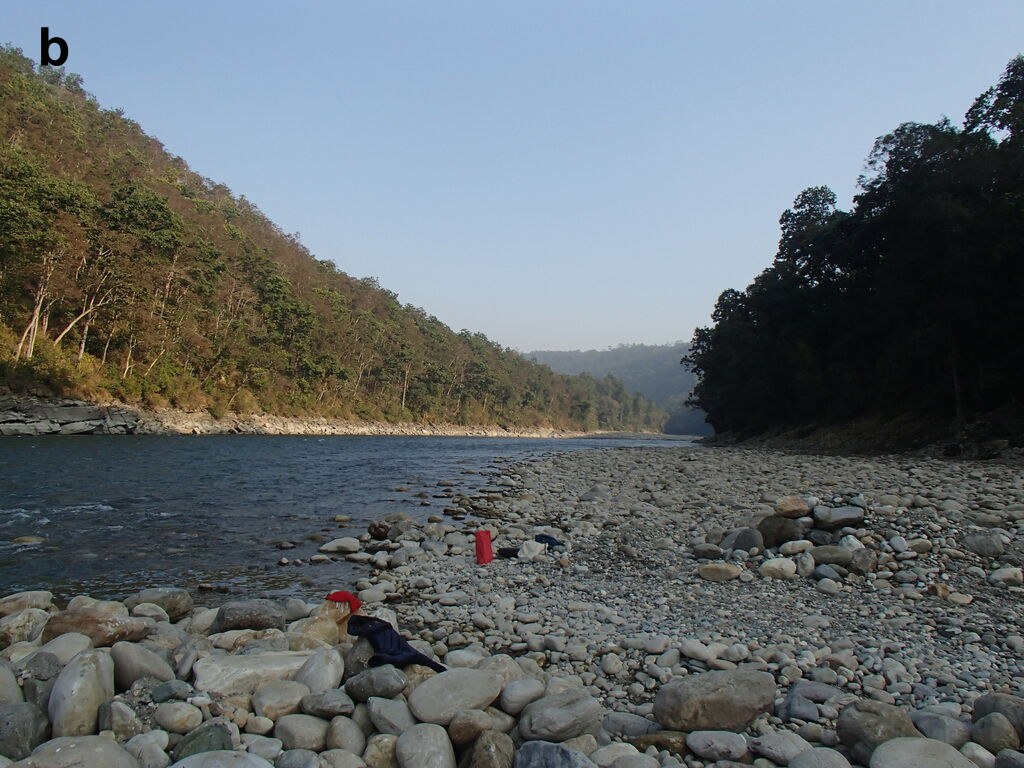
Bheri River (BH2)

**Figure 1c.**
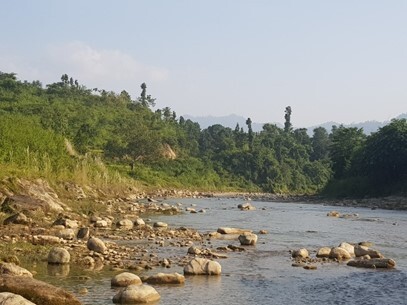
Goche (BHT1)

**Figure 1d.**
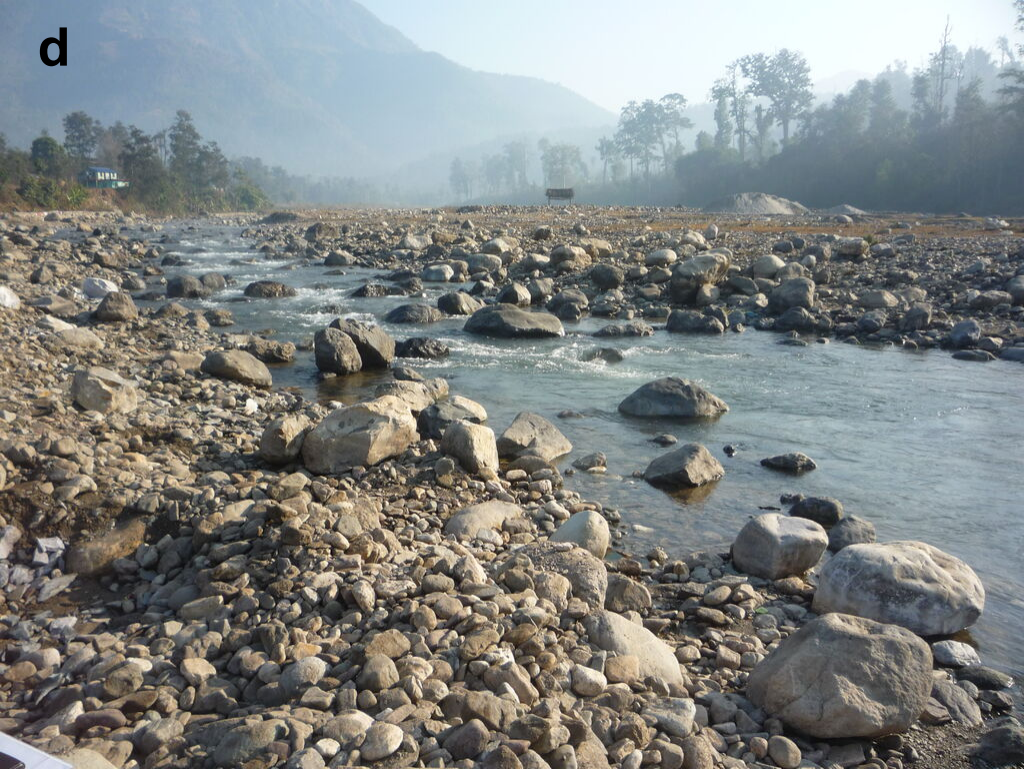
Chingad (BHT2)

**Figure 1e.**
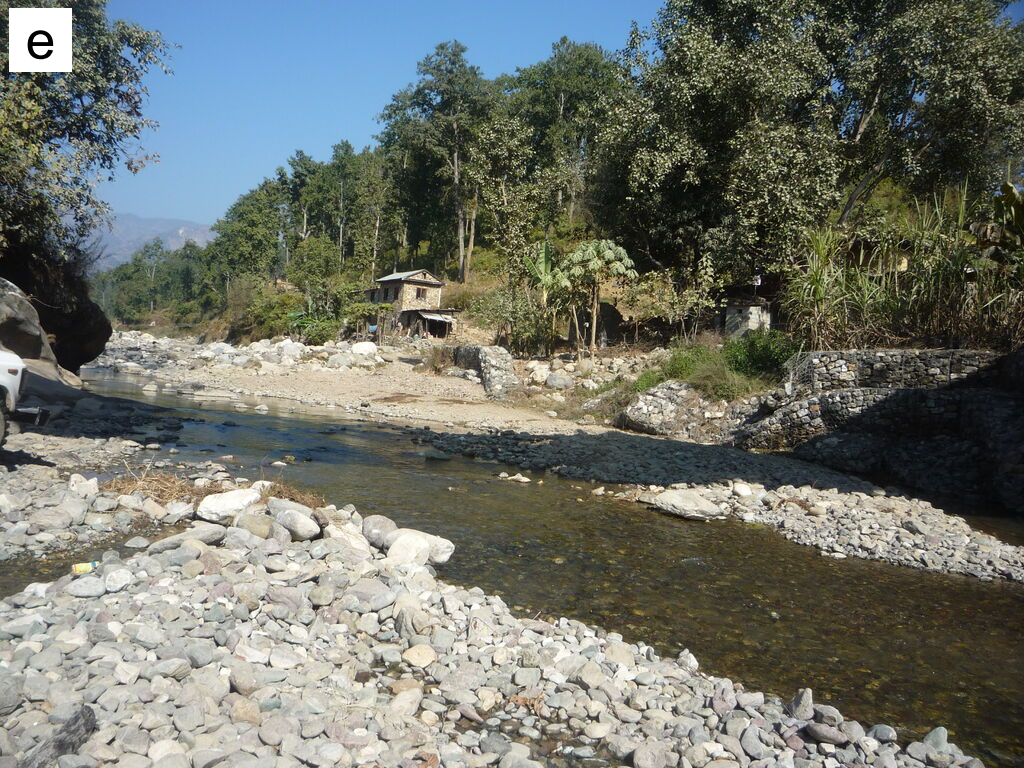
Jhupra (BHT3)

**Figure 1f.**
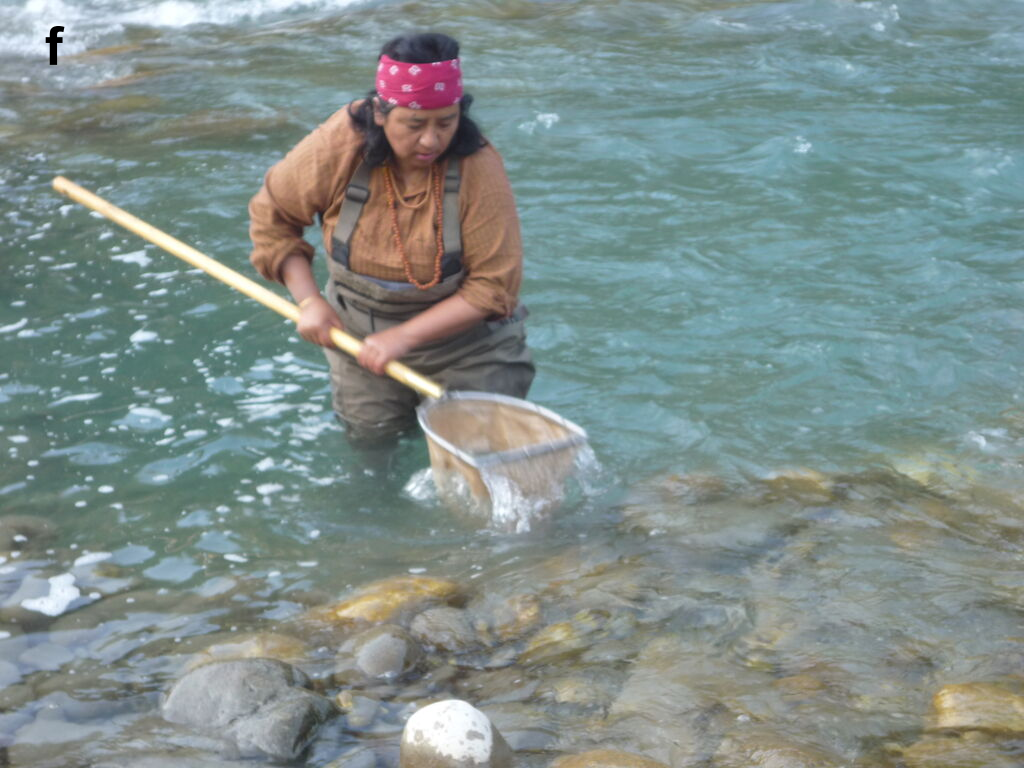
Macroinvertebrate sampling.

**Figure 2a.**
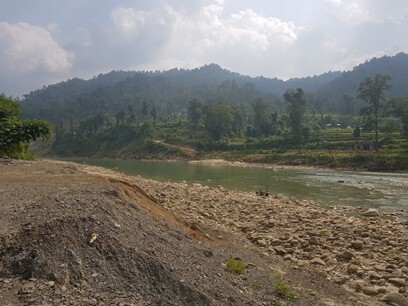
Babai River (BB1)

**Figure 2b.**
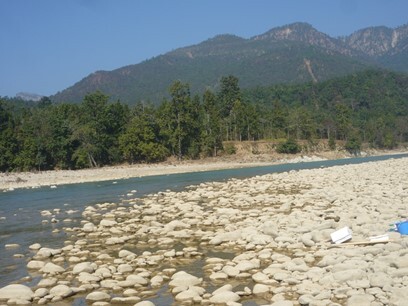
Babai River (BB2)

**Figure 2c.**
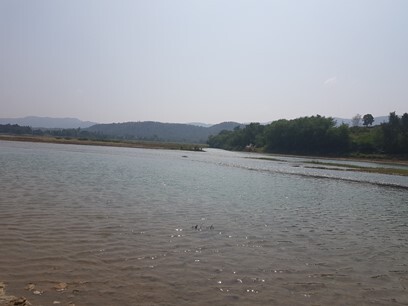
Babai River (BB3)

**Figure 2d.**
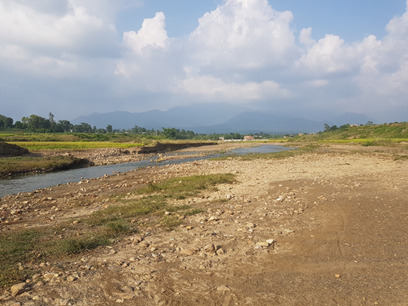
Patre (BBT1)

**Figure 2e.**
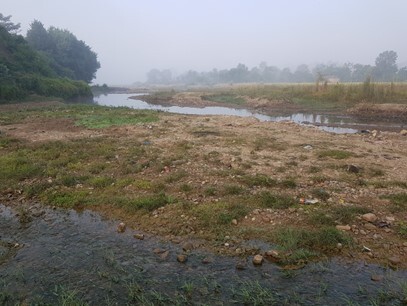
Katuwa (BBT2)

**Figure 2f.**
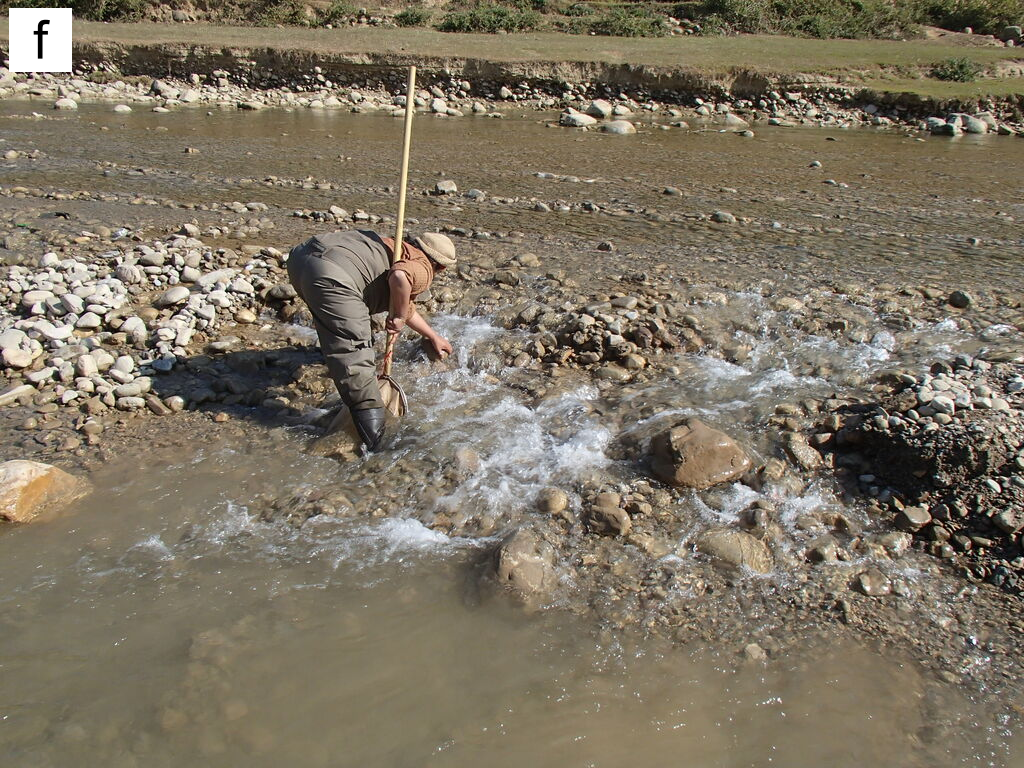
Macroinvertebrate sampling.

**Figure 4a.**
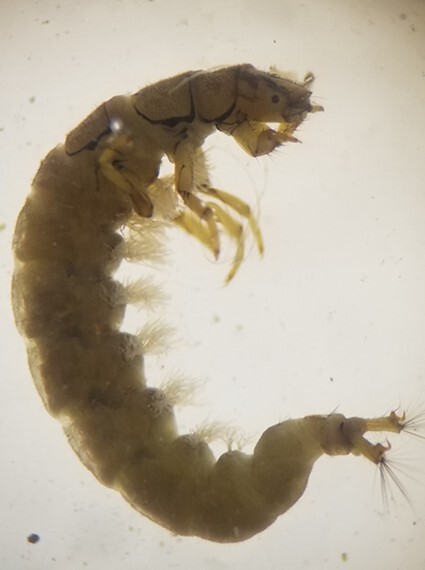
Hydropsychidae

**Figure 4b.**
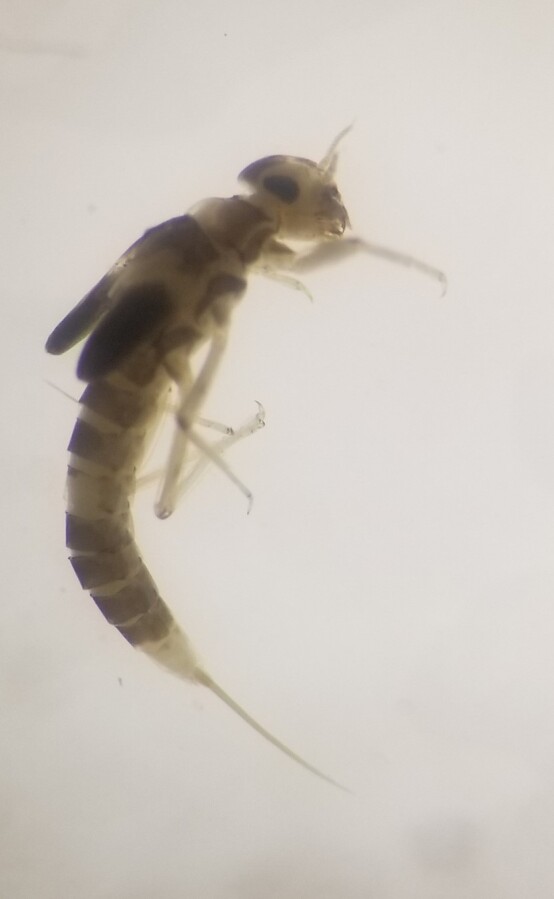
Baetidae

**Figure 4c.**
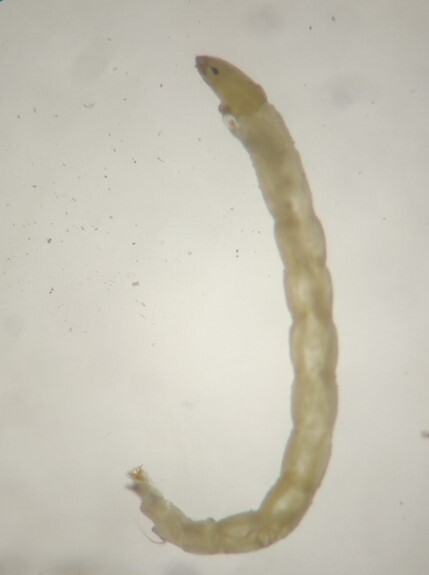
Chironomidae

**Figure 4d.**
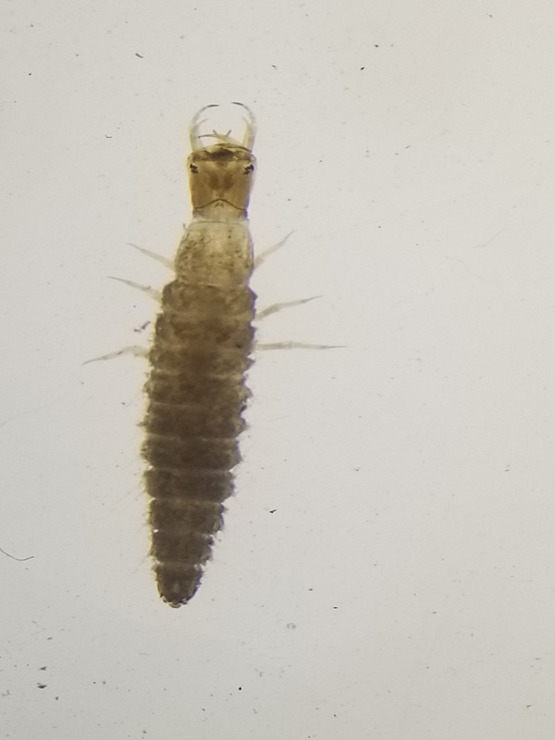
Gyrinidae

**Figure 4e.**
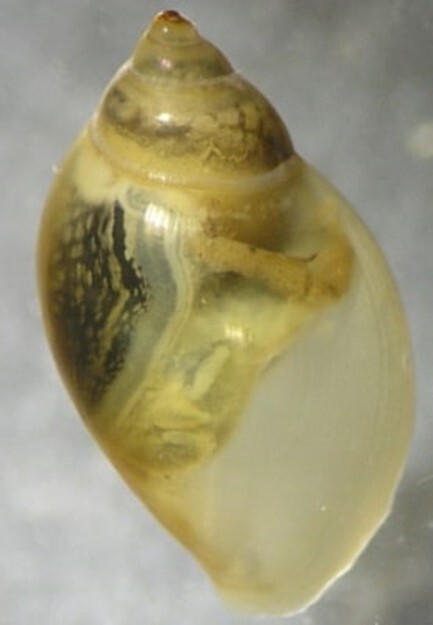
Physidae.

**Table 1.** Sampling sites in the Bheri and the Babai River systems with geographical coordinates and elevation.

**Site Code**	**River**	**Places**	**Elevation** (m a.s.l.)	**Latitude**	**Longitude**	**Remarks**
BH1	Bheri	Surkhet	436	28.45742°N	081.78235°E	Upstream of water diversion at Bheri
BH2	Bheri	Surkhet	403	28.51468°N	081.67520°E	Downstream of water diversion at Bheri
BHT1	Goche	Mehelkuna, Surkhet	475	28.43677°N	081.83489°E	Tributary of Bheri
BHT2	Chingad	Gangate, Surkhet	466	28.55361°N	081.70715°E	Tributary of Bheri
BHT3	Jhupra	Surkhet	497	28.57791°N	081.67207°E	Tributary of Bheri
BB1	Babai	Chepangghat, Surkhet	293	28.35160°N	081.72109°E	Upstream of water release at Babai
BB2	Babai	Mulghat, Bardiya	287	28.36127°N	081.68044°E	Downstream of water release at Babai
BB3	Babai	Bel Takura, Dang	561	28.03095°N	082.26972°E	Upstream of Babai
BBT1	Patre	Majhgaun, Dang	594	28.07607°N	082.37733°E	Tributary of Babai
BBT2	Katuwa	Ghorahi, Dang	625	28.01966°N	082.48380°E	Tributary of Babai

**Table 2.** Status of seasonal macroinvertebrate dominant taxa from the Bheri and the Babai River systems.

River	Season	Dominant taxa	Highest number recorded	Taxa with lowest number
Bheri	Winter	Hydropsychidae	818	Ephemeridae, Veliidae, Elmidae and Glossosomatidae
Bheri	Spring	Baetidae	920	Ephemeridae, Macromiidae, Gyrinidae and Ceratopogonidae
Bheri	Summer	Hydropsychidae	491	Gerridae, Micronectidae and Gyrinidae
Bheri	Autumn	Baetidae	1645	Pyralidae, Ceratopogonidae and Acaridae
Babai	Winter	Gyrinidae	708	Ephemerellidae and Limoniidae
Babai	Spring	Hydropsychidae	580	Calopterygidae, Psephenidae, Philopotamidae and Psychomyiidae
Babai	Summer	Physidae	697	Macromiidae
Babai	Autumn	Chironomidae	679	Heptageniidae, Ephemeridae and Sphaeriidae

**Table 3.** Spatial and temporal variation in species richness and diversity indices.

River	Site	Seasons	Total Number of Orders	Total Number of Families	Shannon-Weiner Diversity (H')	Evenness (J)	Simpson's diversity index (1-D)
Bheri	BH1	Winter	7	17	1.826	0.644	0.745
Bheri	BH2	Winter	5	13	1.451	0.566	0.604
Bheri	BHT1	Winter	10	19	1.020	0.341	0.398
Bheri	BHT2	Winter	5	8	1.874	0.853	0.799
Bheri	BHT3	Winter	4	8	0.641	0.308	0.260
Bheri	BH1	Spring	9	20	2.194	0.721	0.844
Bheri	BH2	Spring	7	22	2.012	0.642	0.800
Bheri	BHT1	Spring	5	8	1.523	0.733	0.738
Bheri	BHT2	Spring	7	14	2.063	0.782	0.840
Bheri	BHT3	Spring	6	13	2.050	0.799	0.839
Bheri	BH1	Summer	5	11	1.892	0.789	0.781
Bheri	BH2	Summer	5	13	1.529	0.596	0.598
Bheri	BHT1	Summer	4	5	0.774	0.481	0.387
Bheri	BHT2	Summer	8	18	1.715	0.593	0.745
Bheri	BHT3	Summer	8	16	2.116	0.763	0.826
Bheri	BH1	Autumn	6	15	2.103	0.776	0.831
Bheri	BH2	Autumn	4	10	1.847	0.802	0.785
Bheri	BHT1	Autumn	6	15	2.241	0.828	0.864
Bheri	BHT2	Autumn	7	16	1.055	0.381	0.398
Bheri	BHT3	Autumn	9	20	1.887	0.620	0.769
**Average**					**1.691**	**0.651**	**0.692**
Babai	BB1	Winter	7	13	1.878	0.732	0.780
Babai	BB2	Winter	7	13	1.625	0.633	0.708
Babai	BB3	Winter	6	8	0.243	0.117	0.079
Babai	BBT1	Winter	8	15	1.334	0.481	0.556
Babai	BBT2	Winter	8	15	1.891	0.698	0.792
Babai	BB1	Spring	6	15	2.209	0.816	0.842
Babai	BB2	Spring	8	19	2.217	0.753	0.832
Babai	BB3	Spring	8	18	1.530	0.529	0.626
Babai	BBT1	Spring	5	10	1.420	0.617	0.644
Babai	BBT2	Spring	2	4	1.134	0.818	0.642
Babai	BB1	Summer	8	13	2.165	0.844	0.837
Babai	BB2	Summer	7	15	1.546	0.571	0.670
Babai	BB3	Summer	10	18	2.447	0.847	0.894
Babai	BBT1	Summer	11	20	2.136	0.713	0.803
Babai	BBT2	Summer	6	11	1.814	0.757	0.774
Babai	BB1	Autumn	7	14	2.162	0.819	0.863
Babai	BB2	Autumn	9	18	1.947	0.674	0.772
Babai	BB3	Autumn	10	24	1.925	0.606	0.809
Babai	BBT1	Autumn	6	10	1.773	0.770	0.796
Babai	BBT2	Autumn	7	14	1.900	0.720	0.764
**Average**					**1.765**	**0.676**	**0.724**

**Table 4.** Transformation table for water quality classification.

NEPBIOS/ASPT Original Scale	NEPBIOS/ASPT for Mid-land	NEPBIOS/ASPT for Lowland	Water quality class
8.00-10.00	7.50-10.00	6.50-10.00	I
7.00-7.99	6.51-7.49	6.00-6.49	I-II
5.50-6.99	5.51-6.50	5.00-5.99	II
4.00-5.49	4.51-5.50	4.00-4.99	II-III
2.50-3.99	3.51-4.50	2.50-3.99	III
1.01-2.49	2.01-3.50	1.01-2.49	III-IV
1	1.00-2.00	1	IV

**Table 5.** Spatial and temporal variation water quality, based on the GRSBIOS/ASPT index.

Season	Winter		Spring		Summer		Autumn	
Site	GRSBIOS/ASPT	WQC	GRSBIOS/ASPT	WQC	GRSBIOS/ASPT	WQC	GRSBIOS/ASPT	WQC
BH1	6.06	II	6.06	II	5.44	II	6.31	II
BH2	6.91	I-II	5.95	II	6.45	II	6.44	II
BHT1	5.89	II	4.57	II-III	4.75	II-III	5.85	II
BHT2	6.25	II	5.92	II	6.13	II	5.80	II
BHT3	6.50	II	5.83	II	6.07	II	6.32	II
BB1	6.25	II	5.54	II	6.15	II	6.15	II
BB2	5.92	II	6.12	II	6.08	II	5.69	II
BB3	6.00	II	5.13	II-III	5.41	II-III	5.52	II
BBT1	4.31	III	4.40	III	5.40	II-III	5.11	II-III
BBT2	4.86	II-III	3.00	III	4.70	II-III	4.58	III
